# Exploring animal behaviour multilayer networks in immersive environments – a conceptual framework

**DOI:** 10.1515/jib-2024-0022

**Published:** 2024-07-24

**Authors:** Stefan Paul Feyer, Bruno Pinaud, Karsten Klein, Etienne Lein, Falk Schreiber

**Affiliations:** Department of Computer and Information Science, 26567 University of Konstanz, Konstanz, Germany; Faculty of Information Technology, Monash University, Clayton, Australia; University of Bordeaux, CNRS, Bordeaux INP, LaBRI, UMR 5800, Bordeaux, France; Behavioural Evolution Lab, Max Planck Institute of Animal Behavior, Konstanz, Germany

**Keywords:** multilayer networks, animal behaviour, network visualisation, immersive analytics, software concept

## Abstract

Animal behaviour is often modelled as networks, where, for example, the nodes are individuals of a group and the edges represent behaviour within this group. Different types of behaviours or behavioural categories are then modelled as different yet connected networks which form a multilayer network. Recent developments show the potential and benefit of multilayer networks for animal behaviour research as well as the potential benefit of stereoscopic 3D immersive environments for the interactive visualisation, exploration and analysis of animal behaviour multilayer networks. However, so far animal behaviour research is mainly supported by libraries or software on 2D desktops. Here, we explore the domain-specific requirements for (stereoscopic) 3D environments. Based on those requirements, we provide a proof of concept to visualise, explore and analyse animal behaviour multilayer networks in immersive environments.

## 1 Introduction

Networks play an important role in the Life Sciences for data exploration and analysis, e.g., in knowledge graphs [1], biological networks [2] or social structures [3]. These networks model entities and their relation to other entities.

If an entity has more than one type of relation to other entities, multilayer networks can be considered, where each type of relation is explicitly addressed in a separate layer. The additional layers open new avenues for modelling complex systems where the complexity of relationships is better embraced as several interdependent subsystems or layers rather than a simple graph. The notion of layers is turned into an actionable apparatus used to better model questions and hypotheses [4, 5]. A multilayer network is thus a network with subnetworks represented by layers.

In the field of animal behaviour, while networks and network analysis are well-established research methods [3], multilayer networks have only recently been used. In 2018, Silk et al. raised the question “Can multilayer networks advance animal behaviour research?” [6], and since then various examples positively answer this question [7–15]. For example, Sharma et al. [10] found that “[…] the potential of an individual wasp to become a queen only was revealed, when social interaction in four different situations were considered simultaneously”, and thus gained an insight with multilayer networks that would not have been gained with mono-layer networks, compare Figure 2.

While multilayer networks are useful for characterising behavioural phenotypes, their utility extends beyond conventional network analysis. One application lies in exploring group cohesion dynamics. By incorporating various aspects such as social rank, spatial proximity, speed or acceleration into different layers, multilayer networks can capture complex patterns of social interactions and allow, for instance, the detection of leadership positions or tracing the flow of influence through social networks [16]. Understanding how interaction rates or specific behaviours scale with spatial proximities between individuals can offer valuable insights into the social dynamics of aggregations or stable social groups. In this context, multilayer networks present a promising avenue to the study of territorial behaviour. Additionally, when social groups aggregate into larger colonies, as is, for instance, the case in some species of cichlid fishes, certain individuals appear to be more prone to defend their group against neighbouring factions. By visualising such territorial defence mechanisms within a multilayer network, the complex dynamics of group defence become more discernible, allowing for insights into social roles. Furthermore, the stability of hierarchies and relationships within social groups is a common area of scientific interest, but also with practical implications concerning sampling methodologies. Representing, for instance, different sampling periods in separate layers of a multilayer network would enable researchers to assess the stability of social ties over time, or alternatively, uncover unsuitable observation periods. The application of multilayer network thus not only enhances our understanding of social structures and their dynamics, but importantly, also provides a useful tool to scrutinise the validity and representativeness of sampled data.

Thus far, the majority of studies have used traditional 2D screen desktop applications like muxViz [17] for the visualisation and analysis of multilayer networks. However, McGee et al. [18] see potential in 3D and immersive network representations, especially with recent technological developments and research [19]. We also believe that immersive and interactive environments for the visualisation and analysis of animal behaviour multilayer networks could be beneficial to the community.

In this paper, we provide a concept for the visualisation, exploration and analysis of animal behaviour multilayer networks in immersive stereoscopic 3D environments such as virtual reality (VR) or augmented reality (AR). Furthermore, we describe the part of the design space of multilayer networks which is spanned by layer dimensionality and layer arrangement.

The paper is structured as follows: After this introduction, we provide an overview of related work on this topic in Section 2. In Section 3 we discuss the requirements and design considerations of the concept and how this concept could be realised. We provide a potential usage scenario in Section 4, and close with some remaining challenges and a conclusion in Section 5.

## 2 Related work on multilayer networks

First, we start with the definition of multilayer networks. A multilayer network *M* = (*V*_*M*_, *E*_*M*_, *V*, *L*) consists of finite sets of nodes *V* and layers *L*. *V*_*M*_ is a subset of all node-layer combinations with *V*_*M*_ ⊆ {(*v*, *l*)∣(*v*, *l*) ∈ *V* × *L*}, and *E* is a subset of all edges between those node-layer combinations with *E*_*M*_ ⊆ {((*v*, *l*), (*v*′, *l*′))∣(*v*, *l*), (*v*′, *l*′) ∈ *V*_*M*_ × *V*_*M*_}. An edge is defined as an *edge between layers* if *l* ≠ *l*′, and as an *edge on a layer* if *l* = *l*′.

There are various terms and definitions that partially describe extensions and variations but partially also describe the same concepts, see [18, 20]. Notable related concepts are *heterogeneous networks* (in particular heterogeneous networks on multiple levels [21]), *overlapping networks* [22] and to some extent *clustered graphs* [23] and *typed* or *attributed networks* [24]. For example, every multilayer network can also be seen as a heterogeneous network on multiple levels.

Finn et al. [7] identify two main multilayer network types: (a) *multiplex networks* (compare Figure 4) and (b) *interconnected networks* (compare Figure 5). In *multiplex networks*, each layer comprises the same set of nodes (e.g., individuals) and edges between layers connect the node to itself on other layers. Subcategories are *multirelational networks*, where each layer represents a different type of interaction, and *temporal networks*, where each layer represents a time step. In *interconnected networks*, each layer can hold different nodes and edges between layers can connect different nodes. Subcategories are *networks of networks* which consists of subsystems that are networks themselves, *intercontextual networks* where each layer represents a different type of nodes and *spatial networks* which are networks of locations.

### 2.1 Visualisation of multilayer networks

For visual analytics approaches, multilayer networks need to be visualised properly. The design space for traditional networks is vast and has been extensively explored, as evidenced by established models and techniques [25]. In addition to this already extensive design space, multilayer networks introduce an additional dimension, primarily characterised by the integration of multiple layers.

**Figure 1:**
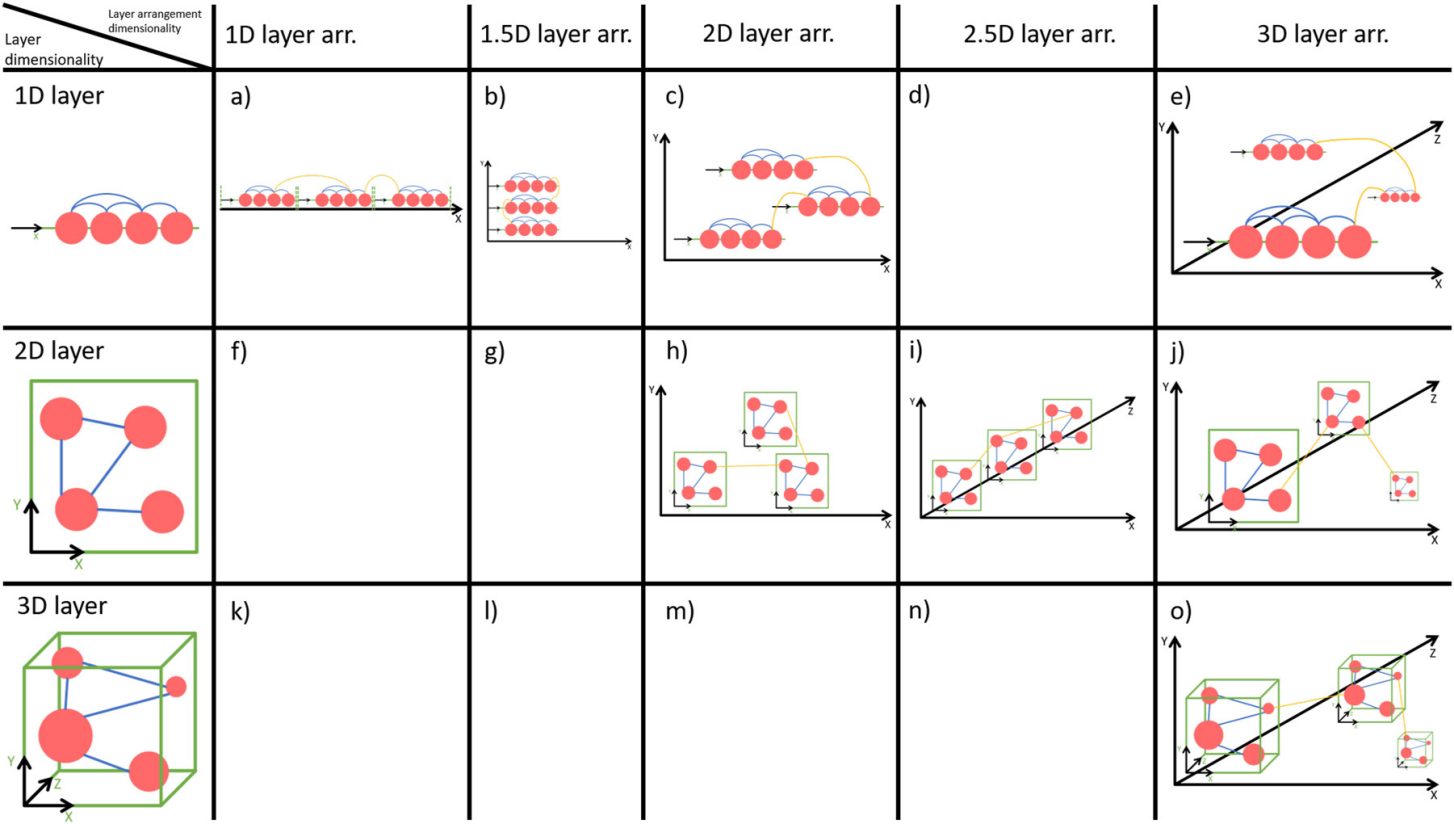
Part of the design space of multilayer network visualisation, spanned by 1D, 2D and 3D layers, and 1D, 1.5D, 2D, 2.5D and 3D layer arrangements.

A single layer can be drawn in different ways, especially in different dimensions. On a one-dimensional (1D) layer, all nodes are aligned on a line along one coordinate axis, often edges are allowed to be in 2D, compare the 1st row (1D layer) of Figure 1. A 2D layer is the most common layer dimensionality, the nodes are aligned in 2D space along two coordinate axes, see 2nd row of Figure 1. In a 3D layer, nodes are located in 3D space and aligned along three coordinate axes, compare 3rd row of Figure 1. 3D layers are less common and outside stereoscopic viewing conditions, there is no empirical evidence for the benefits of 3D network visualisations [18].

In addition to the layer dimensionality, layers can be arranged in different ways, simply called *layer arrangement* or sometimes *embedding*. In a 1D layer arrangement, layers are arranged along one coordinate axis, see 1st column of Figure 1. In a 2D layer arrangement, layers are arranged along two coordinate axes, see 3rd column of Figure 1. In a 3D layer arrangement, layers are arranged along three coordinate axes, see 5th column of Figure 1. In the 2.5D layer arrangement, layers are stacked above each other utilising the third dimension with restrictions, compare the 4th column of Figure 1. The fields below the diagonal are not listed due to layer dimensionality exceeds the dimensionality of the layer arrangement, field (d) is a special case of field (b). A recent study of 2D and 3D layers in 2D, 2.5D and 3D layer arrangement revealed that layer arrangements (h), (I) and (o) are suitable depending on the visual analysis task considered [19].

**Figure 2:**
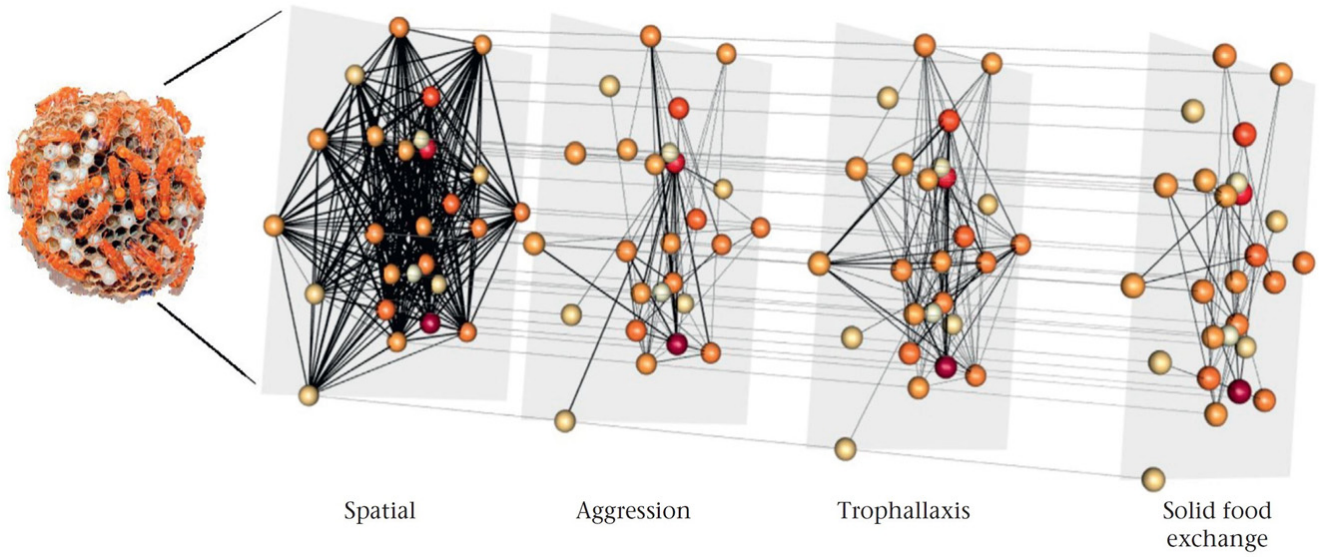
Example of a multiplex network with 4 layers in 2.5D layer arrangement with edges between the layers. The layers represent four behaviours: spatial, aggression, trophallaxis and solid food exchange. Image taken from Sharma et al. [10] (published open access under licence CC BY-NC-ND 4.0 DEED).

There are many visualisation approaches for multilayer networks and related concepts such as heterogeneous networks and stacked networks in general. Here, we focus on the application domain of animal behaviour and the usage of visualisation approaches within this application. The common visualisations of animal behaviour multilayer networks in literature mostly utilise 2D layers. The layers are shown as individual networks side-by-side without the edges between the layers [10, 11, 15], often with additional information encoded in node visualisation and edge visualisation [13, 14, 26, 27]. In Ref. [12] these layers are shown in perspective as 2.5D layer arrangement without the edges between layers. The authors in Ref. [10] show their network in 2.5D with edges between the layers (compare Figure 2), and in Ref. [14] 1D layers are used in a 1.5D layer arrangement for visualising pedigree data. Matrix visualisations for multilayer networks were introduced by Finn [8] in 2021 but are to our knowledge very seldom used (e.g., in publications). The above-mentioned literature reveals, that the most present multilayer network type in animal behaviour is the multiplex network with 2D layers in the 2.5D layer arrangement.

Since a network with 2D layers in a 2.5D layer arrangement is inherently a 3D object, it is a logical step to bring those networks into stereoscopic 3D viewing conditions, such as augmented reality (AR) or virtual reality (VR) and immersive environments, to use of the potential benefits. An *immersive environment* refers to a space designed to fully engage the senses and create a feeling of being surrounded by and involved in an experience. This often involves technologies like virtual reality or augmented reality to create environments that users can interact with in a highly immersive manner. Immersive environments support *immersive analytics* [28–30] by using immersive technologies to analyse and visualise data in a way that allows users to interact with and explore complex datasets more intuitively and effectively. By leveraging immersive technologies, users can dive into data visualisations, manipulate data representations, and explore correlations and patterns in a more immersive and interactive manner than traditional 2D visualisations. This approach has the potential to enhance data understanding, decision-making, and collaboration among analysts and other stakeholders [31]. Furthermore, there is initial evidence, that stereoscopic 3D visualisations of mono-layer networks are beneficial in some occasions [32–34] and we believe this outcome could translate to multilayer networks, too.

To our knowledge, the visual analytics of multilayer networks primarily focuses on providing an overview, with the analytics component largely driven by algorithms. However, we perceive an opportunity for enhancing the visual analytics of multilayer networks through immersive analytics within immersive environments, if the tool fulfils certain criteria: user-friendliness, integration of visualisation with real-time metric extraction capabilities, and a reduction in visual clutter. Given the inherently complex nature of multilayer networks, the importance of interactive functionality is crucial. In this regard, immersive environment applications offer promising solutions.

### 2.2 Visualisation software

There are several software programs and libraries for the visualisation and analysis of multilayer networks and related network types such as stacked networks and heterogeneous networks. WilmaScope1
https://wilma.sourceforge.net/. [35] and GEOMI [36] are early 3D graph visualisation systems that can be used for visualising stacked or multilayer networks as shown in Refs. [22, 37], respectively. Pymnet2
http://www.mkivela.com/pymnet/. [38] and multiNetX3
https://pypi.org/project/multinetx/. [39] are Python libraries, Multinet4
https://rdrr.io/cran/multinet/. [40] and muxViz5
https://muxviz.wordpress.com/info/. [17] are libraries for R. VERTIGo6
https://github.com/erickedu85/vertigo. [41] is a visual platform for querying and exploring large multilayer networks. Arena3D [42] visualises networks in the biological domain using interactive 3D layouts. Other visualisation approaches and life science applications for multilayer network are also discussed in Refs. [21, 43].

Tools for the visualisation, exploration and analysis of multilayer networks in stereoscopic 3D are less common. Arena3Dweb7
https://arena3d.org/. [44] is a version of Arena3D as web application, with a comparable scope and functionality. By using a web browser and a VR head-mounted display (HMD), one can look at a static visualisation of multilayer networks, without the possibility to interact with the network. MNET-VR8
http://leonardmaxim.com/mnetvr/. [45] is a tool for the exploration of multilayer networks in VR. GAV-VR9
https://zenodo.org/records/8289512. [46] was used in Ref. [19] for a study on multilayer network visualisations in VR.

Up to our knowledge, there are no software programs with a comparable capability in stereoscopic 3D such as augmented or virtual reality. Moreover, we are not aware of any software tailored to the needs of animal behaviour researchers for visualising, exploring and analysing animal behaviour multilayer networks. Considering the increasing importance of multilayer networks in the field of animal behaviour, we believe that the work presented in this paper will be beneficial to the community.

## 3 Requirements and design considerations

McGee et al. [18] see the potential for 3D and immersive multilayer network representations and Silk, Finn and Hasenjager et al. [6–9] described the use of multilayer network analysis in animal behaviour. Based on this background, we propose a concept for a system for visualising, exploring and analysing multilayer networks in immersive environments. The elements that shape our concept are described below.

### 3.1 Analysis of animal behaviour multilayer networks

Jones et al. [14] collected data from 14 populations of caribou and generated two networks based on this data: pedigree and spatial co-occurrence. They combined these two networks into one multilayer network and stated “The use of multilayer analysis allows us to assess how the connections in one context (genetic relatedness) relate to the connections in another (spatial co-occurrence) in a single framework”. One of the central measures they used is edge overlap, which represents how often an individual is connected across all layers. Sharma et al. [11] tracked griffon vultures with GPS and generated four layers out of the spatial data. The main analysis methods they used were layer aggregation and centrality measures.

In Ref. [7], it is specifically discussed how multilayer networks can enhance the understanding of an individual’s role in a social network as well as group-level structure and dynamics, population structure and evolutionary models of the emergence of sociality. Additionally, they provide potential research aims:

– Identifying important or influential nodes or edges, e.g., “How will a group be affected if a certain individual will be removed?”,
– Quantifying network properties at different scales e.g., “What are the coherent groups in a network of animals?”,
– Model statistical properties of a network e.g., “are interaction patterns influenced by group size?”
– Modelling disease or information transmission e.g., “what influences disease transmission in multispecies communities?”

In the context of animal behaviour, networks are characterised by the frequency of interactions, their intensity and duration. For instance, aggressive behaviours can vary in occurrence and the duration of each instance requires layers that capture both aspects to draw conclusions. Additionally, animal behaviour networks are often more volatile, highlighting the need for dynamic network visualisation. Researchers often create these networks by observing one animal at a time, which results in a combined view of interactions that don’t happen all at once. Such artefacts must be considered in the visualisation and analytical processes to ensure accurate representation and interpretation. A common visualisation approach to achieve this is node and edge appearance, compare Section 2.1.

Wey et al. [3], Finn et al. [7, 8], Hasenjager et al. [9] and Silk et al. [6] define crucial analysis methods for the analysis of animal behaviour multilayer networks. Together with additional methods found in literature (compare Section 2), we provide in Table 1 a condensed list of analysis methods, that a system for the analysis of animal behaviour multilayer networks should provide to effectively support researchers.

**Table 1:** Common methods for the analysis of animal behaviour multilayer networks and their description.

Method	Description
Eigenvector versatility	Measure of how versatile a node is based on its connectivity within the MLN
Betweenness versatility	Measure of how often a node lies on the shortest path between other pairs of nodes of the MLN
Multidegree	Generalisation of node degree to account for multiple layers in a MLN
Multislice modularity maximisation	Detecting community structure in MLNs by optimising a modularity function
Stochastic block models	Statistical model of arbitrary block structures in MLNs
Motifs	Subgraphs that occur more frequently in a network than expected by chance
Global overlap	Measure of how much nodes and edges overlap between layers in a multilayer network
Randomisation	Generating null models by shuffling edges while preserving certain properties of the MLN
ERGM (exponential random graph models)	Statistical models used to analyse the structure of MLNs and their formation processes
Markov models of coevolving multiplex models	Models that describe the dynamics of MLNs as Markov processes
Stochastic actor-oriented models for multiple networks	Models used to analyse how actor attributes and network structure influence network dynamics
Compartmental models on networks	Models that describe the dynamics of disease spreading or information diffusion on networks
Layer aggregation	Combining information from multiple layers of a multilayer network into a single representation
Outdegree centrality and versatility	Measure of the number of outgoing edges from a node in each layer of the MLN, along with the variation or diversity of outdegree centrality across layers
Outstrength centrality and versatility	Similar to outdegree centrality, but considers the sum of weights on outgoing edges instead of just counting the number of edges

In addition to providing a comprehensive suite of predefined methods, the system should offer ease of extensibility for integrating new custom methods that arise from specific research questions. This ensures seamless integration of problem-tailored analysis methods into the system. Furthermore, the system must be user-friendly and accessible, accommodating a wide range of immersive environment hardware setups while ensuring a positive user experience.

### 3.2 Operations on multilayer networks

Tools and libraries for the visualisation, exploration and analysis of networks such as Vanted [47], Tulip [48], Cytoscape [49] and OGDF [50] all have in common that they provide a basic set of operations to interact with networks (e.g., add nodes/edges, load and store network, resize/colour nodes/edges). These operations have to be provided by our system, too. Additionally, more operations are necessary for multilayer networks:

**Colour Mapping:** Customised node and edge appearance tailored to animal behaviour multilayer networks, such as sex-based colouring, edge thickness based on overlap, and centrality-based node colouring.
**Separate Layer:** Allows users to define a new layer by selecting a subset of nodes.
**Merge Layer:** Enables the grouping of two or more layers into one cohesive layer.
**Node Layout Algorithms:** Assigns positions to nodes in space, considering the cardinality of multiple layers.
**Layer Layout Algorithms:** Arranges layers on e.g., 2D, 2.5D or 3D for optimal visualisation.
**Move Layer:** Translates a layer in space while preserving the layer arrangement.
**Layer Distance:** Adjusts the distance between layers without disrupting the layer arrangement.
**Filter Layers:** Identifies layers based on specific measures for focused analysis.
**MLN Generator:** Generations of multilayer networks based on user-defined parameters.
**Hide and Show Layers:** Allows users to toggle the visibility of layers and corresponding edges.
**Transform Layers:** Adjusts the size and shape of layers for enhanced visualisation.
**Sort Layers:** Organises layers based on measures within the layer arrangement for improved clarity.

### 3.3 Data and data formats

The underlying animal behaviour data of multilayer networks is collected by researchers in-lab or in-field. The next step is then to transform the collected data into multilayer networks. For this process, often external tools are utilised like in Refs. [13, 14]. The multilayer network then needs to be transformed into file formats that are supported by the analysis tool. For example, MuxViz uses edge list as input, MultiNet uses its own data format.10
https://rdrr.io/cran/multinet/manIO.html, accessed 30.04.2024. Another common data format is GraphML.11
https://docs.opennms.com/horizon/32/development/topology/graphml.html#layers, accessed 30.04.2024. Thus, the system for visualising, exploring and analysing animal behaviour multilayer networks should support all common file types used to store multilayer networks.

The number of nodes, layers and edges as well as properties mapped to the appearance of nodes and edges depends on the collected data and the research question.

In literature, one node often represents one individual, thus the size of those networks relates to the group size of the observed data. Jones et al. [14] studied 14 populations with 50–300 individuals but state that a population are in most cases < 100 individuals. Per population, they generated a multilayer network with two layers with various R packages. Sharma et al. [11] studied 29 individuals on four layers. Bonnell et al. [26] studied three troops of monkeys with around 20 individuals on four layers.

### 3.4 Technical realisation

The realisation of our concept for visualising, exploring and analysing animal behaviour multilayer networks could be a standalone implementation or an extension of an already existing tool. GAV-VR [46] is a framework for network analysis and visualisation in virtual reality. As such, GAV-VR already provides the basic functionality for mono-layer networks. Because it is a framework that is easy to extend, GAV-VR is a good starting point to realise our proposed concept.

**Figure 3:**
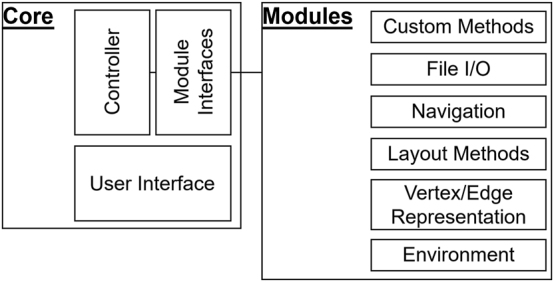
Structure of GAV-VR comprising the *Core* and the *Modules*. Taken from Ref. [46], published open access under CC BY 4.0 licence.

Figure 3 shows the basic structure of the GAV-VR framework divided into two main parts: *Core* and *Modules*. *Core* provides the core functionalities and manages the modular features. It provides a user interface (UI), that allows the analyst to interact with the networks with operations like loading a network, node and edge appearance and location and transforming a network. Furthermore, the core of GAV-VR can handle a large variety of VR head-mounted displays, which makes GAV-VR usable with any hardware supported by SteamVR.

**Figure 4:**
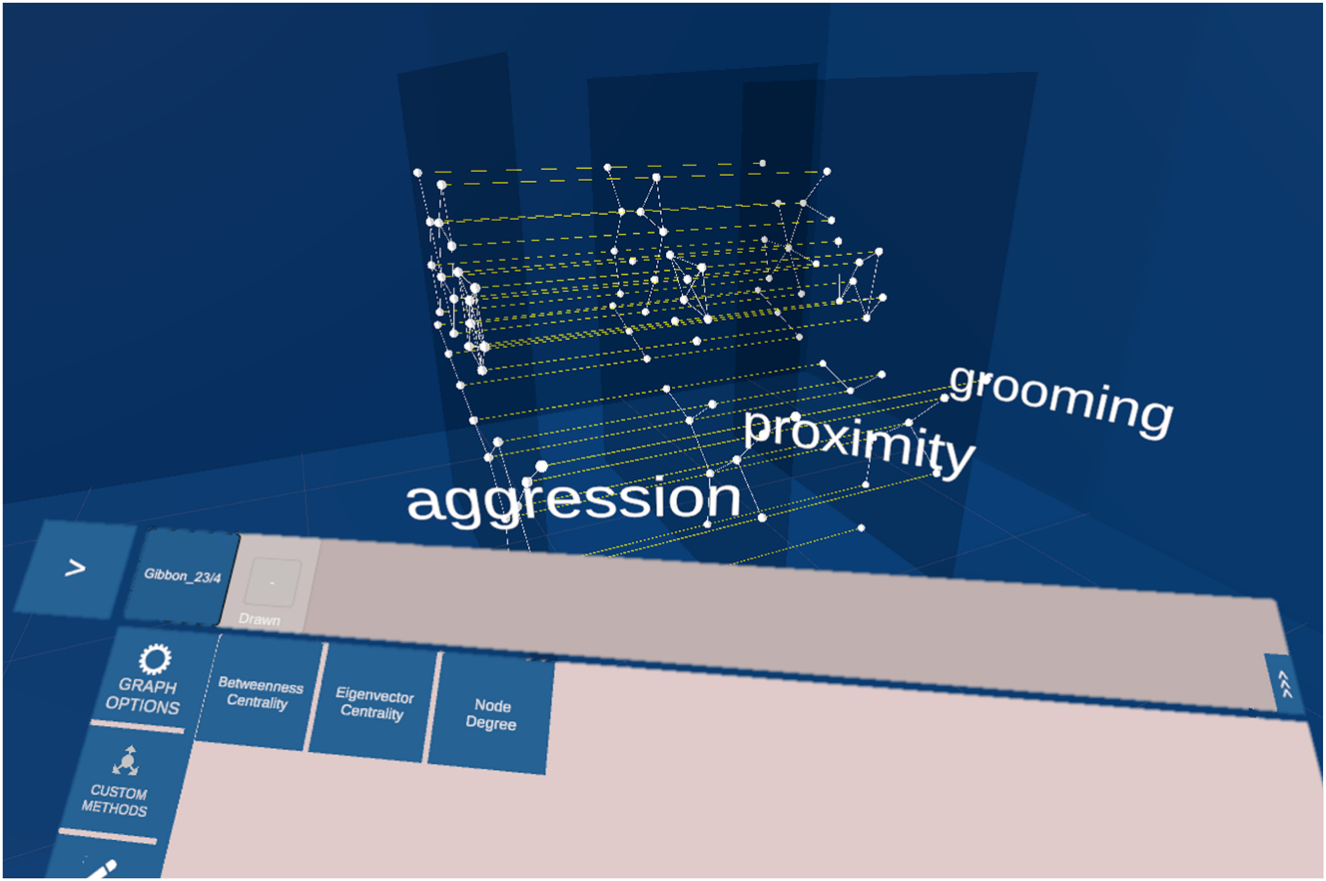
Mockup of a multiplex network with 3 layers. Each layer holds the same set of nodes. The edges on the layer represent the behaviour: proximity, aggression and grooming. The edges between the layers connect the same individuals. In the lower part of the image the panel used to interact with the network is located. In the *custom methods* tab are 3 analysis methods listed: *betweenness centrality*, *eigenvector centrality* and *node degree*.

To meet the requirement defined in the previous section for a tool for visualising, exploring and analysing animal behaviour multilayer networks some extensions need to be implemented as modules. A File I*/*O module handles the various data types to read and parse from any file format, and a layout method module contains layout methods for proper visualisation of *interconnected networks* and *multiplex networks*. The standard analysis methods can be implemented as *Custom Methods* module, which automatically appear in the user interface (UI), compare Figure 4 bottom. The analyst needs the possibility to change the appearance of nodes and edges interactively and link analysis values or properties to the appearance, which can be realised in a *Vertex/Edge Representation* module.

**Figure 5:**
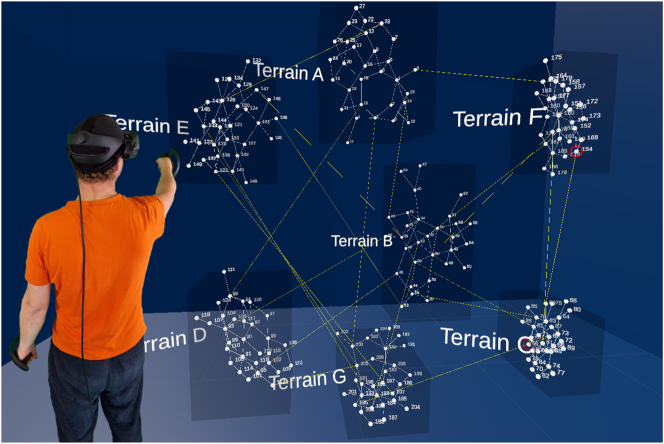
Mockup of interconnected network with 7 layers representing different terrains and nodes representing individuals. A researcher interacts with the networks with controllers in hand.

## 4 User scenario

A researcher observed in-field a population of 20 individuals. The observation was focussed on three behaviours: *aggression*, *proximity* and *grooming*. This results in a multiplex network with three layers, on each layer are 20 nodes representing each individual. The researcher loads the network into the system, and the system automatically uses the correct parser and visualises the network. In the default 2.5D layer arrangement, the labels beside each layer identify the interaction type visualised by the edges on this layer, compare Figure 4. First, the researcher colours the nodes representing females in one colour and males in another. Then the researcher colours the edges and maps the thickness of the edges to the weight of the edges. Because some areas of the network are cluttered, the researcher applies a layout algorithm which considers all layers and adjusts the distance between the layers. To observe each layer individually, the researcher grabs one layer after another with the controller and moves it out of the network. By grabbing the layer with both controllers and pulling them apart, the layer scales in size. To put the layer back into its arrangement, the researcher places it back into the layer arrangement where the layer snaps into position. The researcher is interested in the betweenness versatility of the individuals, thus the researcher navigates on the UI to the methods panel and chooses the corresponding method. The values are visualised beside the nodes. To make the values more visible, the researcher colours the nodes with a linear colour mapping to the betweenness versatility value. In the next step, the researcher is interested in how the values change when some layers are aggregated. For this, the researcher selects the layers of interest and clicks on the *merge button*. To separate the layers again, the researcher selects the layers and clicks on *separate*. Additionally, the researcher is interested in how the values change if only male or female individuals are considered. In the filter tab, the researcher first hides the females and shows them again, and then the males. Because of the improved vision, the researcher decides to create two multilayer networks, one with the males and one with the females. To do so, the researcher selects the subset of nodes and clicks on *create new network*. The researcher is pleased by the outcome and stores the networks to share it with the community.

## 5 Future work and conclusion

In recent years, multilayer networks has gained more and more importance in the field of animal behaviour, and several researchers state the benefit of multilayer networks for specific research questions in this area. Those research questions need a set of analysis methods, visualisation techniques and tools. While those tools exist for general multilayer networks, there is no tool to specifically support the analysis of animal behaviour multilayer networks. In addition, there is no tool capable of multilayer networks analysis and visualisation in immersive environments. We provided an in-detail analysis of which methods are necessary, which tools exist and what additional operation must be added to mono-layer network analysis. We presented a detailed concept that facilitates the visualisation of animal behaviour multilayer networks in immersive environments. Implementing this concept will significantly enhance the capabilities of researchers in this field.

The implementation of the proposed concept is currently underway as an ongoing project. Current challenges include the technical limitations of the scalability and complexity for large multilayer networks. Furthermore, developing efficient internal data representations of multilayer networks as well as suitable encodings are crucial. Mapping research questions to analysis process steps and their resulting design implications is a major challenge.

With input from the research community, there is potential to gain deeper insights into workflows and develop concepts to support these workflows better. A usability study could prove the suitability of the implemented concept. With the implemented concept in hand, layout algorithms and exploration algorithms tailored to the needs of immersive analytics in immersive environments can be created and evaluated.

One potential for enhancement is the integration of 2D desktop applications like muxViz, allowing for a seamless transition to the immersive environment (so-called transitional or hybrid interfaces [51–54]), or integrating these tools directly into the immersive environment.
